# The interplay of reward responsiveness and socioeconomic disadvantage in the prospective prediction of depression symptoms in youth

**DOI:** 10.1017/S0033291725102729

**Published:** 2025-12-03

**Authors:** Christine Roberts, Cope Feurer, Alexandra Petryczenko, Maria Granros, Katie L. Burkhouse

**Affiliations:** 1 The Research Institute, Nationwide Children’s Hospital, USA; 2 Department of Psychiatry, The University of North Carolina at Chapel Hill, USA; 3 Department of Psychology, Penn State University: The Pennsylvania State University, USA; 4 Department of Psychiatry, University of Illinois Chicago, USA

**Keywords:** area deprivation index, depression, event-related potential, reward positivity, socioeconomic disadvantage, electroencephalography, income-to-needs, youth, reward responsiveness, stress exposure

## Abstract

**Background:**

Alterations in reward responsiveness represent a key mechanism implicated in youth depression risk. However, not all youth with these alterations develop depression, suggesting the presence of factors that may moderate risk patterns. As socioeconomic disadvantage is also related to youth depression risk, particularly for youth exhibiting altered reward function, this study examined whether indices of family- and neighborhood-level disadvantage interacted with electrocortical reward responsivity to predict depression symptom trajectories across childhood and adolescence.

**Methods:**

Participants included 76 youth (ages 9–16 years) at low and high risk for depression based on maternal history of depression. At baseline, youth completed a monetary reward-guessing task while electroencephalography was recorded to measure the reward positivity (RewP), an event-related potential indexing reward responsiveness. Family and neighborhood disadvantage were assessed using the income-to-needs (ITN) ratio and Area Deprivation Index (ADI), respectively. Self-reported and clinician-rated depression symptoms were assessed across a multiwave, 18-month follow-up.

**Results:**

RewP interacted with family- and neighborhood-level disadvantage to predict self-reported depression symptom trajectories. Specifically, blunted RewP predicted self-reported depression symptom increases for youth with a lower ITN ratio and higher ADI score. A blunted RewP also predicted clinician-rated depression symptom increases for youth living in neighborhoods with higher ADI scores.

**Conclusions:**

Findings suggest that reduced reward responsiveness is a mechanism implicated in future depression risk among youth, specifically in the context of family- and neighborhood-level socioeconomic disadvantage. Interventions that enhance reward response among youth exposed to higher levels of socioeconomic disadvantage may be particularly effective in preventing depression emergence.

## Introduction

Major depressive disorder (MDD) is one of the most common psychological disorders, with late childhood to adolescence representing a developmental period of increased risk for first onset of this disorder (Hammen, 2009; Wilson & Dumornay, 2022). Accordingly, ~20% of youth reported experiencing a depressive episode in the past year (NIMH, 2023). This is particularly important as the onset of MDD in childhood is associated with higher rates of depression (Rao & Chen, 2009) and greater severity and chronicity of major depressive episodes (Zisook et al., 2007) in adulthood, and it puts individuals at increased risk for suicide (Gould et al., 2003) and substance abuse problems (Lai et al., 2015). Considering adolescence as a critical period of heightened risk, it is crucial to identify specific risk factors linked to depression onset to inform prevention efforts. Therefore, the present study seeks to identify biopsychosocial risk factors that may conjointly enhance risk for depression during adolescence.

Altered positive valence system functioning is a well-established correlate of depression (Kujawa et al., 2020). For instance, across studies, youth depression risk is associated with diminished reward processing, as observed across neural (e.g. electroencephalography [EEG], functional magnetic resonance imaging) and behavioral units of analysis (for a review, see Kujawa & Burkhouse, 2017). One neural index of reward processing that is linked with depression risk is the reward positivity (RewP), which is an EEG-derived event-related potential (ERP) component that reflects neural reactivity to consumption of rewards (Proudfit, 2015). Blunted RewP has been identified as a biomarker for depression, with studies showing that a reduced RewP predicts increases in symptoms (Bress et al., 2013; Kujawa et al., 2019; Panier et al., 2024) and diagnoses (Belden et al., 2016; Michelini et al., 2021; Nelson et al., 2016) of depression in children and adolescents.

While research highlights blunted RewP as an established risk marker for depression, there may be subgroups of individuals for whom this risk factor is most pronounced (for a review, see Burkhouse & Kujawa, 2023). One potential risk factor that may exacerbate the impact of blunted reward processing on depression risk is stress exposure. Previous studies have found that stress exposure (e.g. acute, social, and cumulative life stress) is associated with greater levels of depression symptoms (Lemoult et al., 2020), especially in youth exhibiting a reduced RewP (Burani et al., 2021; Goldstein et al., 2020; Pegg et al., 2019). We have also previously shown that greater family-level financial stress during the coronavirus disease 2019 (COVID-19) pandemic interacted with reduced RewP to predict mid-pandemic increases in depression symptoms (Feurer et al., 2021). Together, these prior works support a diathesis-stress model of depression risk, suggesting that reduced neural reward responsiveness interacts with higher stress exposure to predict depressive symptoms in youth.

In the present study, we sought to extend our prior study (Feurer et al., 2021), testing this diathesis-stress model of depression risk with a longer, multiwave follow-up period (18 months) to assess trajectories of depression change, spanning beyond the most acute period of the COVID-19 pandemic. Additionally, we sought to expand on these previous findings by examining additional indices of socioeconomic disadvantage that may predict unique variance in youth depression risk. Prior research examining how the interplay between RewP and stress exposure relates to depression has predominantly focused on stress exposure at the individual or family level (Burani et al., 2021; Goldstein et al., 2020). However, youth exist within larger contexts, and research shows that neighborhood-level factors, such as neighborhood-level socioeconomic disadvantage, influence youth development and well-being (Bronfenbrenner, 1977). Therefore, rather than just looking at socioeconomic disadvantage at the family level, there has been increasing emphasis in recent years to also consider neighborhood-level disadvantage, which predicts unique variance in youth neurodevelopment and psychopathology risk that is independent of the influence of family-level disadvantage (Barch, 2022). For example, not only is neighborhood disadvantage linked with depression risk (Cutrona et al., 2006; Richardson et al., 2015; Sui et al., 2022), but this association is independent of the influence of family-level socioeconomic status (Galea et al., 2007; Xue et al., 2005). Similar effects have been observed when considering the impact of socioeconomic disadvantage on neurodevelopment, such that neighborhood-level disadvantage predicts unique variance in brain structure and function when statistically controlling for family-level socioeconomic status (Miller et al., 2022; Ramphal et al., 2020; Whittle et al., 2017). Therefore, it is important to examine how both family- and neighborhood-level disadvantage may interact with RewP to predict unique variance in youth depression risk.

To explore these relationships, we examined whether RewP interacted with commonly assessed family-level (i.e. income-to-needs [ITN] ratio) and neighborhood-level (i.e. area deprivation index [ADI]) indices of socioeconomic disadvantage to predict trajectories of depression symptoms among a sample of youth enriched for depression risk. Youth depression symptoms were assessed across a multiwave follow-up period (i.e. baseline, 6, 12, and 18 months) to assess symptom trajectories. To capture a comprehensive picture of depressive symptomatology, we assessed both self-reported and clinician-rated symptoms. Self-report measures provide direct access to individuals’ subjective internal experiences, whereas clinician-rated assessments offer a standardized, observer-based evaluation of symptom severity and functional impairment, helping to reduce potential self-report bias. Including both perspectives allows us to test whether the interactive effects of neurophysiological reward processing (i.e., RewP) and socioeconomic disadvantage are robust across informant and assessment methods, thereby strengthening the validity and generalizability of our findings. Consistent with prior research showing that stress exposure exacerbates the impact of blunted RewP on youth depression symptom increases (Feurer et al., 2021; Murry et al., 2011; Nelson et al., 2016; Xue et al., 2005), we hypothesized that increases in depression symptoms would be most pronounced for youth exhibiting a blunted RewP and experiencing increased levels of family- and neighborhood-level socioeconomic disadvantage.

## Methods

### Participants

Participants were from two larger longitudinal studies examining the intergenerational transmission of depression between mothers and their children, recruited between 2017 and 2022. Specifically, Study 1 examined mother–daughter dyads with and without a maternal history of MDD to examine mechanisms in the intergenerational transmission of depression and included female youth between the ages of 11 and 16 years. Study 2 focused on a preventative intervention for depression in male and female children between the ages of 9 and 15 years at high risk (HR) for depression (i.e. with a maternal history of MDD). As the focus of this analysis was to examine predictors of depression symptom trajectories in youth, participants from Study 2 were only included in the current sample if they were part of the control group that did not participate in the intervention. The current sample consisted of 76 youth (86.8% female) between the ages of 9 and 16 years (mean age = 13.50, standard deviation [SD] = 2.23). Regarding racial identity, 61.8% of youth identified as White (42.1% as non-Latiné), 10.5% as Black, 13.2% as Asian, 7.9% as multiracial, and 6.6% as another racial identity. Additionally, 31.6% identified as Latiné. Data collection for both studies occurred at the same site. To assess the history of any DSM-5 (American Psychiatric Association, 2022) psychiatric disorders and to confirm study eligibility, trained research assistants and master- or doctoral-level clinicians administered diagnostic interviews to mothers and youth under the supervision of a licensed clinical psychologist. Specifically, youth and mothers completed the Schedule for Affective Disorders and Schizophrenia for School Age Children (Kaufman et al., 1997), and mothers completed the Structured Clinical Interview for DSM-5 (First et al., 2015). Both studies recruited mother–child dyads based on mothers’ history of MDD. Specifically, mothers in the low-risk (LR) dyads (*n* = 27) had no lifetime history of depression, whereas mothers in the HR dyads (*n* = 49) had a lifetime history of MDD (*n* = 38 recurrent MDD; *n* = 6 current MDD). Mothers and youth were excluded for neurological disorders; history of a traumatic brain injury; intellectual or developmental disability; active suicidal ideation; history of bipolar spectrum disorder, psychosis, or schizophrenia; and diagnosis of an alcohol or substance use disorder in the past 6 months. Mothers in the LR group were also excluded if they endorsed any lifetime history of DSM-5 psychiatric disorders. Additionally, youth were excluded if taking any psychotropic medications at the time of study enrollment, and meeting criteria for a history of bipolar, schizophrenia, or conduct disorder. Finally, given the focus of the larger studies on prospective prediction of depression or prevention of depression, youth were excluded from Study 1 if they had any lifetime history of MDD and from Study 2 if they had a current diagnosis of MDD.

Of the youth in the study, the following mood disorders were endorsed: lifetime MDD (HR, *n* = 1) and lifetime disruptive mood dysregulation disorder (HR, *n* = 1). In addition, the following anxiety disorders were endorsed: lifetime generalized anxiety disorder (HR, *n* = 1), current generalized anxiety disorder (HR, *n* = 8; LR, *n* = 2), current social anxiety disorder (HR, *n* = 4), current specific phobia (HR, *n* = 2), current obsessive-compulsive disorder (HR, *n* = 1), lifetime obsessive-compulsive disorder (HR, *n* = 1), and lifetime post-traumatic stress disorder (HR, *n* = 1). Finally, the following other disorders were endorsed: current attention-deficit hyperactivity disorder (ADHD) inattentive type (HR, *n* = 3), current ADHD combined type (HR, *n* = 2), current oppositional defiant disorder (HR, *n* = 1), and lifetime oppositional defiant disorder (HR, *n* = 1).

The following other disorders were endorsed by the mothers in the HR group: alcohol use disorder (lifetime, *n* = 1), anorexia nervosa (lifetime, *n* = 1), binge-eating disorder (lifetime, *n* = 3; current, *n* = 1), panic disorder (lifetime, *n* = 3; current, *n* = 1), agoraphobia (lifetime, *n* = 1; current, *n* = 1), social anxiety disorder (lifetime, *n* = 8; current, *n* = 9), generalized anxiety disorder (lifetime, *n* = 8; current, *n* = 6), specific phobia (lifetime, *n* = 1; current, *n* = 1), obsessive-compulsive disorder (lifetime, *n* = 3; current, *n* = 2), and post-traumatic stress disorder (lifetime, *n* = 17; current, *n* = 1).

### Depression symptoms

Youth depression symptoms were assessed at baseline and across follow-ups (6, 12, and 18 months) using self-report (Center for Epidemiologic Studies Depression Scale [CESD]; Radloff, 1977) and clinician-administered (Children’s Depression Rating Scale–Revised [CDRS-R]; Poznanski et al., 1984) measures. The CESD is a 20-item self-report measure that assesses depression symptoms in the past week. CESD scores can range from 0 to 60, and higher scores on the CESD indicate greater symptom severity. The CDRS-R is a clinician-administered 17-item measure that assesses depression symptoms in the past 2 weeks. The summary score (interviewer-derived consensus among parent and child scores) was used in analyses. Given that the CDRS-R was administered to some families virtually during the COVID-19 pandemic, observational items 15–17 were omitted from the total score. Therefore, the total possible scores for this modified CDRS-R ranged from 14 to 94. See Table 1 for information about the distribution of CESD and CDRS-R scores across all time points.

**Table 1.** Descriptive statistics and correlations for the main study variables

	1.	2.	3.	4.	5.	6.	7.	8.	9.	10.	11.	12.	13.	14.	15.
1. Study	–														
2. Maternal MDD	.50^***^	–													
3. Child baseline age	−.50^***^	−.48^***^	–												
4. Child sex (% female)	−.57^***^	−.29*	.37^**^	–											
5. Child race/ethnicity (% Non-Hispanic White)	−.01	.02	−.03	−.06	–										
6. Income-to-needs ratio	−.19	−.08	.02	.02	.29*	–									
7. Area Deprivation Index	.15	.05	−.18	−.01	−.37^***^	−.57^***^	–								
8. RewP	.14	.16	.15	−.02	.11	−.002	−.14	–							
9. CESD – Baseline	.14	.20	−.02	−.06	−.12	−.16	.003	.17	–						
10. CESD – Follow-up 1	−.08	.29*	.01	.003	−.12	.06	−.08	.002	.36^**^	–					
11. CESD – Follow-up 2	−.03	.30*	.07	.13	−.05	−.14	.08	.03	.40^***^	.59^***^	–				
12. CESD – Follow-up 3	.22	.45^***^	−.05	.11	−.22	−.41^***^	.19	−.05	.38^**^	.43^***^	.68^***^	–			
13. CDRS-R – Baseline	.13	.25*	.03	−.30*	−.09	.04	−.15	−.02	.35^**^	.21	.20	.35^**^	–		
14. CDRS-R – Follow-up 2	−.01	.20	.19	.13	−.19	−.17	.06	−.11	.14	.33^**^	.61^***^	.62^***^	.26*	–	
15. CDRS-R – Follow-up 3	.10	.33^**^	−.02	.04	−.10	−.17	.002	−.13	.21	.18	.46^***^	.60^***^	.49^***^	.38^**^	–
**Mean (SD)**	31.6%	64.5%	13.51 (2.23)	86.8%	42.1%	3.36 (1.36)	31.04 (23.07)	0.00 (6.99)	12.70 (7.67)	12.86 (7.88)	12.74 (9.36)	14.37 (10.75)	18.39 (4.58)	18.93 (6.66)	18.56 (6.21)
**Range**	–	–	9.10–16.97	–	–	0.09–6.60	5–99	−17.16 – 18.78	0–33	0–32	0–34	0–52	14–37	14–48	14–41

*Note*: Study = Study which participants participated in, coded as Study 1 = 0, Study 2 = 1. MDD, major depressive disorder; RewP, reward positivity; CESD, Center for Epidemiologic Studies Depression Scale; CDRS-R, Children’s Depression Rating Scale – Revised.

*p < .05; ^**^p < .01; ^***^p < .001.

### Family-level disadvantage

An ITN ratio was calculated based on self-reported household income when families enrolled in the study, divided by the federal poverty level for their family size for the year they enrolled.

### Neighborhood-level disadvantage

To assess neighborhood-level disadvantage, the 2019 ADI was used. The ADI is a nationally normed factor score indexing neighborhood disadvantage derived using 5-year census data from the American Community Survey (ACS). Factor scores are derived from 17 ACS indicators that assess education, employment, income, and housing quality. The ADI defines neighborhoods as census block groups, and ADI scores were based on the youth’s current address at the time of study enrollment. ADI scores range from 1 to 100 and represent the level of neighborhood disadvantage compared to the national scale. Higher ADI scores are indicative of greater neighborhood-level disadvantage.

### Doors reward task (RewP)

To assess youth RewP, youth completed the Doors Reward Task (Mackin et al., 2021; Proudfit, 2015; see Supplementary Figure 1). The Doors Reward Task consisted of showing participants two doors on the computer screen and tasking them to select which door had a prize behind it, using the left and right mouse buttons. After their selection, a fixation cross appeared on the screen for 1,000 ms. Participants were notified if they chose correctly with a green upward arrow or incorrectly with a red downward arrow. The green arrow indicated that they gained $0.50, and the red arrow indicated that they lost $0.25. After the arrow, a fixation mark was presented for 1,500 ms, followed by a screen reading “click for the next round.” The task consisted of 20 gain and 20 loss trials that were presented at random throughout the task. Participants were paid in cash at the end of the appointment for the task.

### EEG data collection and processing

Continuous EEG data were recorded during the Doors task with the ActiveTwo BioSemi system (BioSemi, Amsterdam, The Netherlands). An elastic cap with 34 standard electrode sites, including FCz and Iz, was used on the 10/20 system. One electrode was placed behind each ear on a mastoid. To capture electrooculogram recordings from eye blinks and eye movements, four facial electrodes were used. Two electrodes were placed 1 cm above and below the right eye to measure vertical eye blinks, and two more were placed 1 cm from the outer corner of each eye to measure horizontal eye blinks and movements. Data were then digitized at 24-bit resolution with a cutoff of 1,024 Hz.

Data analyses were conducted using the BrainVision Analyzer 2 software (Brain Products, Gilching, Germany). EEG data were re-referenced offline to the average of the mastoids and band-passed filtered (0.1 Hz high-pass and 30 Hz low-pass). Data were segmented from 200 ms before to 800 ms after reward feedback presentation. Trials were baseline corrected using 200 ms before feedback onset. Eye blink and ocular corrections were completed following standard methods (Gratton et al., 1983). Standard artifact analysis procedures were used to identify the following: a voltage step of >50 μV between sample points, a voltage difference of 300 μV within a segment, and a maximum voltage difference of <0.50 μV within 100-ms intervals. Additionally, each trial was manually inspected, removing individual channels with any remaining artifacts. The RewP was calculated as the average activity 250–350 ms following reward feedback onset at pooled frontocentral electrodes (FCz, Cz, and Fz), where the difference between gain and loss trials was maximal based on visual inspection (Suor et al., 2023). Gain and loss trials were averaged separately. The residual RewP was calculated by regressing ERPs to gain trials onto ERPs to loss trials, saving the unstandardized residual. Greater reward responsivity was indicated by higher/more positive RewP residual values.

### Procedure

Dyads were recruited from the community through flyers, electronic advertisements, e-mail listservs, and social media. After being recruited and screened for eligibility, mother–child dyads provided informed consent and assent, respectively, and their current home address was collected for neighborhood geocoding analyses. At the baseline assessment, youth completed the Doors task (Proudfit, 2015) and were administered the CESD (Radloff, 1977) and CDRS-R (Poznanski et al., 1984). For both studies, participants completed three follow-up assessments that occurred on average 6.13 (SD = 1.52), 11.94 (SD = 2.07), and 18.06 (SD = 2.08) months after baseline. Regarding follow-ups completed, 5.3% of participants completed only one follow-up, 15.8% completed two follow-ups, and 78.9% completed all three follow-ups. The CESD was administered at all three follow-ups, and the CDRS-R was administered at the second and third follow-ups. All study procedures were approved by the Institutional Review Board, and dyads were compensated for their study participation.

## Analytic plan

### Trajectories of self-reported depression symptoms

Linear mixed modeling (LMM) with an autoregressive (AR1) covariance structure was used to examine whether youth RewP and socioeconomic disadvantage moderated trajectories of youth self-reported depression symptoms (i.e. CESD) scores across the follow-up. First, we examined whether family- and neighborhood-level socioeconomic disadvantage interacted individually with youth RewP to moderate youth depression symptom trajectories. Specifically, the main effects of and all two- and 3-way interactions between mean-centered Socioeconomic Disadvantage (family ITN ratio or neighborhood ADI), RewP, and Time (i.e. months since baseline) were entered as fixed effects. Additionally, maternal history of depression (MDD) and Study (i.e. Study 1 vs. Study 2) were mean-centered and included as covariates. Youth CESD scores were the outcome variable, and Intercept and Time were entered as random effects. The Level 1 and Level 2 equations for the primary models were as follows:

**Level 1:**


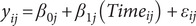



**Level 2:**


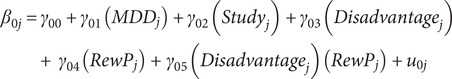




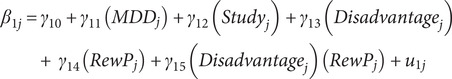



Sensitivity analyses were conducted for significant findings to examine whether findings were maintained controlling for youth age, sex, and racial/ethnic identity (non-Latiné White, yes vs. no). Additionally, if both ITN and ADI individually interacted with RewP to predict youth CESD trajectories, additional follow-up sensitivity analyses were conducted to examine whether both family- and neighborhood-level socioeconomic disadvantage uniquely interacted with youth RewP to predict variance in youth depression symptom changes. This analysis mirrored the LMMs described above, except in this model, all interactions between Disadvantage, RewP, and Time were simultaneously examined for both ITN and ADI in the same model. If ITN and ADI did not both individually interact with RewP to predict symptom trajectories, follow-up sensitivity analyses examined whether findings were maintained when statistically controlling for the other form of socioeconomic disadvantage (e.g., are findings for ITN maintained adjusting for ADI).

### Trajectories of clinician-assessed depression symptoms

Analyses examining whether youth RewP and socioeconomic disadvantage moderated trajectories of youth clinician-assessed depression symptoms (i.e. CDRS-R) across the follow-up largely mirrored analyses focusing on CESD trajectories. However, an inspection of the data revealed that youth CDRS-R scores were significantly and positively skewed across all assessments (baseline *z* = 4.98, follow-up 2 *z* = 8.24, follow-up 3 *z* = 6.07). Therefore, generalized LMM using a gamma distribution with a log link was used instead of LMM to account for the non-normal distribution of the outcome variable.

## Results

See Table 1 for descriptive statistics and correlations among the main study variables. As expected, family ITN and neighborhood ADI were correlated, such that youth whose families had a lower ITN ratio were more likely to live in neighborhoods marked by higher levels of socioeconomic disadvantage. Additionally, as expected based on differences in study eligibility criteria, youth who participated in Study 2 were most likely to be in the HR group (i.e. have a maternal history of MDD), be younger, and be male compared to youth from Study 1. Finally, compared to LR youth, HR youth were more likely to be younger, male, and more likely to exhibit self-reported and clinician-assessed depression symptoms across most time points.

### Trajectories of self-reported depression symptoms

**Figure 1.**
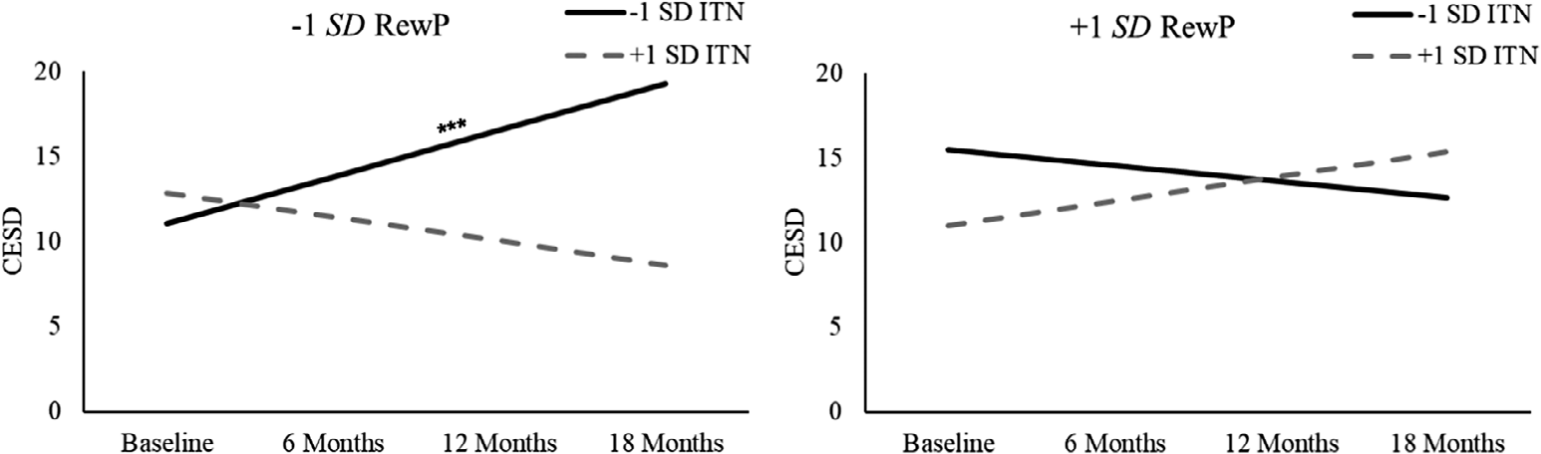
ITN × RewP × Time interaction predicting youth Center for Epidemiologic Studies Depression Scale (CESD) scores across an 18-month follow-up. Simple slopes for Time are presented at high (+1 SD) and low (−1 SD) levels for reward positivity (RewP) and family income-to-needs (ITN) ratio. Significant simple slopes for Time are denoted. ***p < .001.

LMM was used to examine whether decreased RewP moderated trajectories of self-reported depression symptoms (CESD) for youth exposed to socioeconomic disadvantage. See Table 2 for full results. Looking first at the model examining family ITN, there was a significant ITN × RewP × Time interaction, *t*(87.90) = 4.26, *p* < .001. None of the main effects or two-way interactions between ITN, RewP, and Time were significant (lowest *p* = .075). To probe the nature of the ITN × RewP × Time interaction (see Figure 1), simple slopes were tested following procedures by Preacher et al. (2006) to examine the effect of Time on youth CESD at high (+1 SD) and low (−1 SD) levels of RewP and ITN. Consistent with hypotheses, results indicated that youth with a decreased RewP (−1 SD) exhibited statistically significant increases in depression symptoms over time if they also had a lower (−1 SD) family ITN ratio (*t*(74.99) = 4.29, *p* < .001), but not if they had a higher (+1 SD) family ITN ratio (*t*(74.99) = −1.92, *p* = .061). The effect of Time on youth CESD was not significant for youth with an increased (+1 SD) RewP, regardless of whether they had a higher (+1 SD) family ITN ratio (*t*(74.99) = 1.74, *p* = .088) or a lower (−1 SD) family ITN ratio (*t*(74.99) = −1.26, *p* = .215). Finally, sensitivity analyses found that simple slopes for Time remained significant when statistically adjusting for the influence of child age, sex, and racial/ethnic identity (all *p*s < .001).

**Table 2.** Primary linear mixed models examining trajectories of youth CESD

	Income-to-needs				Area Deprivation Index			
Fixed effects	*B*	SE	*t*	*p*	*B*	SE	*t*	*p*
Study	−1.35	2.10	−0.64	.523	−1.36	2.04	−0.67	.507
Maternal MDD	4.01	2.04	1.97	.053	4.05	1.98	2.05	.044
Disadvantage	−0.49	0.63	−0.78	.440	0.01	0.04	0.16	.873
RewP	0.09	0.13	0.75	.453	0.14	0.12	1.18	.240
Time	0.08	0.06	1.29	.199	0.04	0.06	0.58	.564
Study × Time	−0.14	0.15	−0.89	.375	−0.10	0.16	−0.64	.527
Maternal MDD × Time	0.25	0.14	1.84	.070	0.25	0.14	1.75	.085
Disadvantage × RewP	−0.17	0.09	−1.81	.075	0.01	0.01	1.95	.054
Disadvantage × Time	−0.05	0.05	−1.17	.245	0.001	0.003	0.50	.618
RewP × Time	−0.01	0.01	−0.57	.566	−0.01	0.01	−1.34	.183
Disadvantage × RewP × Time	0.03	0.01	4.26	<.001	−0.002	<0.001	−4.45	<.001

*Note*: CESD, Center for Epidemiologic Studies Depression Scale; MDD, major depressive disorder; RewP, reward positivity.

**Figure 2.**
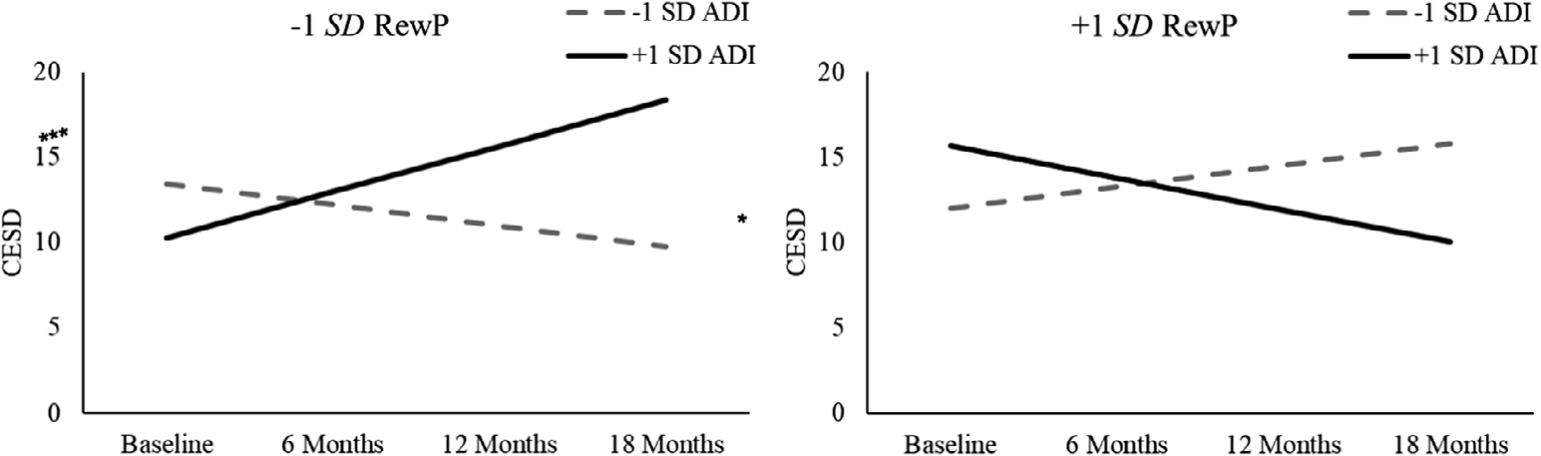
ADI × RewP × Time interaction predicting youth Center for Epidemiologic Studies Depression Scale (CESD) scores across an 18-month follow-up. Simple slopes for Time are presented at high (+1 SD) and low (−1 SD) levels for reward positivity (RewP) and neighborhood Area Deprivation Index (ADI). Significant simple slopes for Time are denoted. *p < .05; ***p < .001.

Looking at the model examining neighborhood ADI (see Table 2), none of the main effects or two-way interactions between ADI, RewP, and Time were significant (lowest *p* = .054). However, similar to findings for ITN, the ADI × RewP × Time interaction was significant (*t*(82.02) = −4.45, *p* < .001; see Figure 2). An examination of simple slopes indicated that youth with a decreased (−1 SD) RewP showed significant increases in depression symptoms over time if they also lived in neighborhoods with a greater (+1 SD) ADI (*t*(76.96) = 4.09, *p* < .001), but not if they lived in neighborhoods with a lower (−1 SD) ADI (*t*(76.96) = −1.56, *p* = .125). Additionally, youth with an increased (+1 SD) RewP exhibited decreases in depression symptoms over time if they lived in neighborhoods with a greater (+1 SD) ADI (*t*(76.96) = −2.14, *p* = .037), but not if they lived in neighborhoods with a lower (−1 SD) ADI (*t*(76.96) = 1.74, *p* = .088). Sensitivity analyses indicated that simple slopes for Time remained significant for youth with decreased RewP and greater neighborhood ADI when statistically controlling for the influence of child age, sex, and racial/ethnic identity (all *p*s < .001). Simple slopes for Time for youth with increased RewP and greater neighborhood ADI remained significant when statistically adjusting for youth sex and race (*p*s ≤ .032), but not age (*p* = .052).

Next, we simultaneously examined the interactions between both ITN and ADI with RewP and Time in the same model to test whether family- and neighborhood-level disadvantage both uniquely interacted with RewP to predict variance in youth trajectories of self-reported depression symptoms. In this model, both the ITN × RewP × Time (*t*(87.77) = 2.09, *p* = .039) and the ADI × RewP × Time (*t*(81.02) = −2.13, *p* = .037) interactions remained significant. Looking first at the unique interaction with ITN, follow-up simple slopes indicated that CESD scores significantly increased across the follow-up for youth with a decreased (−1 SD) RewP who had a lower (−1 SD) family ITN ratio (*t*(75.89) = 2.88, *p* = .006). Simple slopes for Time were not significant for youth with a decreased (−1 SD) RewP and a higher (+1 SD) family ITN ratio, or for youth with an increased (+1 SD) RewP who had either a higher (+1 SD) or a lower (−1 SD) family ITN ratio (all *p*s ≥ .286). Similarly, looking next at the unique interaction with ADI, simple slopes for Time were significant for youth with a decreased (−1 SD) RewP living in neighborhoods characterized by high (+1 SD ADI) socioeconomic disadvantage (*t*(75.89) = 2.11, *p =* .041). Simple slopes for Time were not significant for youth with a decreased (−1 SD) RewP living in neighborhoods characterized by low (−1 SD ADI) socioeconomic disadvantage, or for youth with an increased (+1 SD) RewP living in neighborhoods characterized by either high (+1 SD ADI) or low (−1 SD ADI) socioeconomic disadvantage (all *p*s ≥ .151).

### Change in clinician-assessed depression symptoms

**Figure 3.**
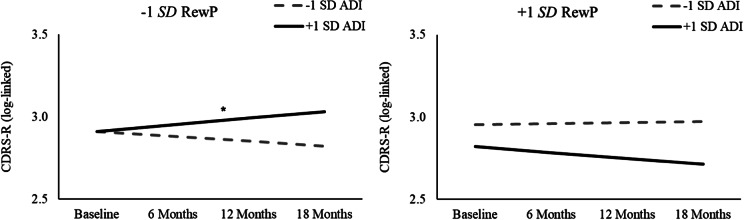
ADI × RewP × Time interaction predicting youth Children’s Depression Rating Scale – Revised (CDRS-R) scores across an 18-month follow-up. Simple slopes for Time are presented at high (+1 SD) and low (−1 SD) levels for reward positivity (RewP) and neighborhood Area Deprivation Index (ADI), and are plotted based on the log-linked regression coefficients from the generalized linear mixed model. Significant simple slopes for Time are denoted. *p < .05.

Generalized linear mixed models were conducted to examine whether exposure to socioeconomic disadvantage moderated trajectories of clinician-assessed depression symptoms (CDRS-R) for youth exhibiting decreased RewP (see Table 3 for full details). In the model testing family ITN, none of the main or interactive effects of ITN, RewP, or Time predicted youth follow-up depression symptoms (lowest *p* = .127). In contrast, in the model testing neighborhood disadvantage, the ADI × RewP × Time interaction was significant (*t*(195) = −2.48, *p* = .014). Follow-up simple slope analyses for the ADI × RewP × Time interaction (see Figure 3) revealed that youth with blunted (−1 SD) RewP exhibited significant increases in CDRS-R over time if they also lived in a neighborhood marked by higher (+1 SD ADI) disadvantage (*t*(195) = 2.07, *p* = .039), but not if they lived in a neighborhood marked by lower (−1 SD ADI) disadvantage (*t*(195) = −1.25, *p* = .212). Simple slopes for Time were not significant for youth with increased (+1 SD) RewP living in neighborhoods with either higher (+1 SD ADI) (*t*(195) = −1.38, *p* = .169) or lower (−1 SD ADI) (*t*(195) = 0.32, *p* = .749) socioeconomic disadvantage. Sensitivity analyses indicated that simple slopes for Time for youth with blunted (−1 SD) RewP living in neighborhoods with a higher (+1 SD) ADI were maintained when statistically controlling for the influence of youth age (*p* = .029), but not sex or racial/ethnic identity (lowest *p =* .073) or family ITN ratio *(p* = .124).

**Table 3.** Primary generalized linear mixed models examining trajectories of youth CDRS-R

	Income-to-needs				Area Deprivation Index			
Fixed effects	*B*	SE	*t*	*p*	*B*	SE	*t*	*p*
Study	−0.02	0.07	−0.31	.757	−0.01	0.07	−0.13	.896
Maternal MDD	0.12	0.07	1.90	.059	0.12	0.07	1.88	.062
Disadvantage	0.01	0.02	0.27	.785	−0.001	0.001	−1.20	.230
RewP	<−0.001	0.004	−0.06	.954	−0.002	0.004	−0.45	.655
Time	<0.001	0.002	0.14	.889	−0.001	0.002	−0.42	.676
Study × Time	−0.002	0.005	−0.42	.678	−0.002	0.01	−0.49	.622
Maternal MDD × Time	0.004	0.004	0.90	.371	0.003	0.004	0.70	.483
Disadvantage × RewP	0.002	0.003	0.83	.407	<−0.001	<0.001	−1.17	.245
Disadvantage × Time	−0.002	0.001	−1.53	.127	<0.001	<0.001	0.59	.556
RewP × Time	<−0.001	<0.001	−0.84	.404	<−0.001	<0.001	−0.85	.394
Disadvantage × RewP × Time	<0.00	<0.001	0.95	.345	<−0.001	<0.001	−2.48	.014

*Note*: CDRS-R, Children’s Depression Rating Scale – Revised; MDD, major depressive disorder; RewP, reward positivity.

Finally, the current study was underpowered to detect four-way interactions with Maternal MDD to examine whether findings were further moderated by Group. However, exploratory analyses found that Disadvantage × RewP × Time interaction was significant for both the family ITN ratio and the neighborhood ADI for self-reported depression symptoms for youth in the HR group. Mirroring primary analyses, simple slopes showed that for HR youth, blunted RewP predicted increases in depression symptoms, but only for youth exposed to higher levels of socioeconomic disadvantage (i.e. low family ITN and high neighborhood ADI). See Supplementary Materials for full results. Moreover, sensitivity analyses were also conducted to examine whether findings for neighborhood disadvantage were maintained when just focusing on neighborhood-level income instead of ADI, to better parallel our family-level indicator of disadvantage. Findings were replicated for the prediction of CESD trajectories, but not CDRS-R trajectories. See Supplementary Materials for results.

## Discussion

This study examined whether neural reward responsiveness and multilevel socioeconomic disadvantage interacted to predict 18-month depression trajectories in youth at LR and HR for the disorder. Consistent with our primary hypothesis, we found that socioeconomic disadvantage moderated the impact of neural reward responsiveness on depression symptom changes among youth. Specifically, when using self-reported depression symptoms as the outcome, findings revealed that youth exhibiting a blunted RewP exhibited a significant increase in their depression symptoms over time if they also lived in families with lower ITN ratios or neighborhoods marked by a higher ADI. Importantly, results indicated that both family- and neighborhood-level disadvantage interacted with blunted RewP to predict unique variance in youth symptom trajectories. We observed a similar effect for clinician-rated symptoms of youth depression with the ADI, but not ITN, such that blunted RewP predicted increases in depression symptoms across the longitudinal follow-up for youth exposed to greater neighborhood disadvantage. Together, these findings support a diathesis-stress model of depression risk, showing that youth exhibiting blunted neural reward responsiveness are at greatest risk for increased depression symptoms if they are exposed to higher levels of socioeconomic disadvantage at both the family and the neighborhood level.

These results are somewhat consistent with the larger literature, which suggests that a blunted RewP is associated with increases in depression diagnoses (Belden et al., 2016; Michelini et al., 2021; Nelson et al., 2016) and symptoms (Bress et al., 2013; Kujawa et al., 2019; Panier et al., 2024). However, the current study extends this work by demonstrating that only those with both a blunted RewP and exposure to higher levels of socioeconomic disadvantage experience increased depression symptoms over time. These results align with the extant literature on the examination of a diathesis-stress model of depression risk (Burani et al., 2021; Feurer et al., 2021; Goldstein et al., 2020; Pegg et al., 2019). For example, Burani et al. (2021) found that only at high levels of stress did an attenuated RewP predict prospective depression symptoms in a sample of peri-adolescent females. Moreover, we previously showed that greater financial stress during the COVID-19 pandemic interacted with blunted RewP to predict increases in depression symptoms (Feurer et al., 2021). The current study extends the previously mentioned findings by showing that blunted reward responsiveness reflects a biological vulnerability to depression, which may be uniquely exacerbated by stressors across family and neighborhood levels, such as socioeconomic disadvantage.

Of note, an unexpecting finding emerged, such that youth with an increased RewP exhibited decreases in depression symptoms over time if they lived in neighborhoods with greater neighborhood disadvantage. One possibility is that youth exhibiting adaptive reward functioning may demonstrate higher prosocial behavior and solicit social support from others in the context of higher stress exposure, which may lead to greater improvement in psychological symptoms (Mancini, 2019). Notably, this finding was not maintained when examining both neighborhood ADI and family disadvantage (ITN) in the same model, and was not observed for clinician-rated symptoms of depression. Thus, further replication is warranted prior to drawing any strong conclusions regarding this unexpected finding.

The current study had multiple strengths, including the assessment of socioeconomic deprivation across family and neighborhood levels and the use of both self-reported and clinician-rated assessments to examine depression symptom trajectories in adolescents. However, the study also had some limitations that warrant discussion. First, the sample size was relatively small, which increases the possibility for exaggerated effect sizes and may contribute to Type I error (Button et al., 2013). Because of the small sample size, we were underpowered to test how maternal depression may have impacted these findings. Given that two-thirds of the sample was enriched for depression risk due to maternal depression history and prior work showing maternal depression interacts with indices of socioeconomic disadvantage to predict offspring RewP (Granros et al., 2024; Israel et al., 2025), future well-powered studies are needed to examine whether the association between socioeconomic disadvantage and attenuated RewP in the prospective prediction of depression is most pronounced among offspring of depressed mothers. Second, because the current sample was drawn from two separate protocols with slightly different inclusion criteria and demographic composition (e.g. sex distribution and prior depression history), there is some possibility that unmeasured differences between studies may have influenced the results, despite the use of identical measures and EEG procedures. Third, as exclusionary criteria for youth included a current diagnosis of MDD at baseline, there may have been limited representation of clinical depression. Future studies should examine whether findings replicate in clinical samples. Fourth, other studies would benefit from including other indices of reward processing, such as reward anticipation or learning, to determine if findings are specific to reward responsiveness or are also observed across other indices of positive valence system functioning. Relatedly, we did not have multiple assessments of family-level socioeconomic status that would allow us to derive a family-level disadvantage factor score to better parallel the established neighborhood ADI factor score. Finally, the addresses used for neighborhood geocoding were collected during consent, and we did not assess whether participants moved over the follow-up. Thus, our measure of ADI did not consider the duration of exposure to the neighborhood or exposure to multiple neighborhoods. This limitation is somewhat alleviated by prior research showing that families tend to move to neighborhoods with similar characteristics (Gorman-Smith et al., 2004). Nonetheless, future studies would be strengthened by multimodal assessment of socioeconomic disadvantage that account for the length of exposure, such as life stress interviews to capture chronic stress exposure (Harkness & Monroe, 2016).

In summary, this study is among the first to examine socioeconomic disadvantage at both the family and neighborhood level in conjunction with youth’s neural reward responsiveness to predict depression symptom changes across adolescence. Our findings support a diathesis-stress model of depression risk, such that youth exhibiting reduced neural reward responsiveness are at greater risk for depression if they are exposed to higher levels of socioeconomic disadvantage. Ultimately, the current findings may lead to targeted prevention programs for youth depression. For instance, prevention programs aimed at enhancing positive affect and reward responsiveness in youth experiencing increased levels of socioeconomic deprivation may prove to be useful in preventing the emergence of depression. We previously showed the utility of a mother–child dyadic preventative intervention targeting positive valence system functioning via upregulating positive affect in youth with a maternal history of depression (Burkhouse et al., 2023). In our pilot study, only youth in the intervention group, not the control group, demonstrated increases in positive affect and reductions in perceived stress and internalizing symptoms. Thus, future work would benefit from examining whether a similar prevention program would be effective in reducing the emergence of depression in youth exposed to higher levels of socioeconomic disadvantage.

## Supporting information

Roberts et al. supplementary material Roberts et al. supplementary material

## Data Availability

The data that support the findings of this study are available from the corresponding author upon reasonable request.

## Supplementary material

The supplementary material for this article can be found at http://doi.org/10.1017/S0033291725102729.
