# Supplementary material for: The interplay of reward responsiveness and socioeconomic disadvantage in the prospective prediction of depression symptoms in youth
Source: Psychol Med. 2025 Dec 3;55:e369. doi: 10.1017/S0033291725102729 (PMC12887541; doi:10.1017/S0033291725102729)

**Supplementary Materials**

**Exploratory Analyses Among HR Youth**

Exploratory analyses were conducted to examine whether findings were maintained when looking within the HR subgroup. Exploratory analyses focused on the HR subgroup (n = 49), and not the LR subgroup (n = 27), given power concerns about testing a 3-way interaction in the LR group.

***Results for CESD***

Mirroring primary findings for CESD, the ITN × RewP × Time interaction was significant, *t*(50.91) = 3.12, *p* = .003. Additionally, simple slopes indicated that the main effect of Time was positive for youth exhibiting blunted (-1 *SD*) RewP who also had a lower (-1 *SD*) family ITN ratio, *t*(46.57) = 4.02, *p* < .001, but not if they had a higher (+1 *SD*) family ITN ratio, *t*(46.57) = -1.14, *p* = .265. The effect of Time on youth CESD was not significant for youth with an increased (+1 *SD*) RewP, regardless of whether they had a higher (+1 *SD*) family ITN ratio, *t*(46.57) = 1.39, *p* = .173, or a lower (-1 *SD*) family ITN ratio, *t*(46.57) = -0.45, *p* = .654.

Similarly, the ADI × RewP × Time interaction was also significant in the HR subgroup, *t*(49.33) = -4.12, *p* < .001. Simple slopes analysis indicated that youth with a decreased (-1 *SD*) RewP showed significant increases in depression symptoms over time if they also lived in neighborhoods with a greater (+1 *SD*) ADI, *t*(46.93) = 4.34, *p* < .001, but not if they lived in neighborhoods with a lower (-1 *SD*) ADI, *t*(46.93) = -1.08, *p* = .293. However, youth did not show any significant changes in depression symptoms over time if they had an increased (+1 *SD*) RewP and lived in neighborhoods with either a greater (+1 *SD*) ADI, *t*(46.93) = -1.75, *p* = .089, or a lower (-1 *SD*) ADI, *t*(46.93) = 1.79, *p* = .084.

***Results for CDRS-R***

Looking at the prediction of clinician-rated depression symptoms, the ITN × RewP × Time interaction was not significant, *t*(122) = 1.43, *p* = .157. The ADI × RewP × Time interaction was significant, *t*(122) = -2.18, *p* = .031. However, none of the simple slopes for Time were significant for youth exhibiting increased (+1 *SD*) or decreased (-1 *SD*) RewP living in neighborhoods marked by higher (+1 *SD*) or lower (-1 *SD*) neighborhood ADI (all *p*s > .153.).

**Neighborhood Median Income Sensitivity Analyses**

Supplemental sensitivity analyses were conducted to examine whether findings for neighborhood disadvantage were maintained when examining neighborhood median income instead of the larger ADI factor score. Mirroring the ADI score, median neighborhood income estimates (aggregated at the block group level) were taken from the 2019 American Community Survey 5-Year data. Median neighborhood income estimates were not available from the ACS for 4 participants from this sample due to their block group having an insufficient number of sample observations.

***Results for CESD***

Consistent with primary results using the larger neighborhood ADI score, supplementary analyses focusing on neighborhood median income revealed a significant Neighborhood Income × RewP × Time interaction, *t*(76.33) = 2.87, *p* = .005. Additionally, simple slopes analysis indicated that youth with a decreased (-1 *SD*) RewP showed significant increases in depression symptoms over time if they also lived in neighborhoods with a lower (-1 *SD*) neighborhood median income, *t*(75.07) = 3.13, *p* = .003, but not if they lived in neighborhoods with a higher (+1 *SD*) neighborhood income, *t*(75.07) = -0.08, *p* = .940. Youth did not show any significant changes in depression symptoms over time if they had an increased (+1 *SD*) RewP and lived in neighborhoods with either a higher (+1 *SD*), *t*(75.07) = 0.98, *p* = .335, or a lower (-1 *SD*), *t*(75.07) = -1.57, *p* = .124, neighborhood median income.

***Results for CDRS-R***

The Neighborhood Income × RewP × Time interaction was not significant in the model predicting youth CDRS-R scores, *t*(185) = 1.90, *p* = .059.

*Figure 1. Schematic of the doors reward task.*


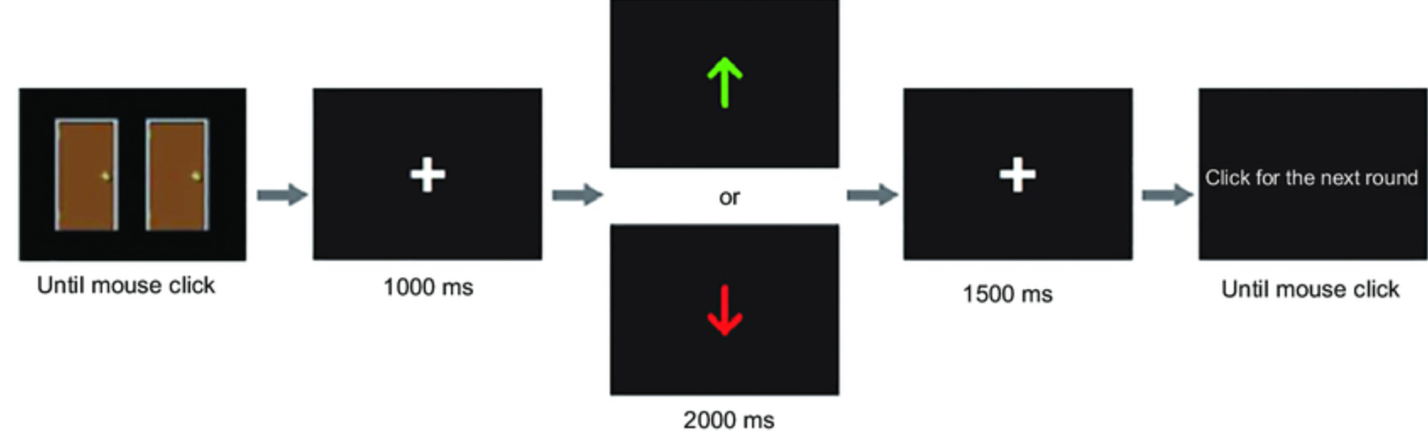

Supplement: Roberts et al. supplementary material [file S0033291725102729sup001.docx]
